# Supplementary material for: Peptidoglycan recycling mediated by an ABC transporter in the plant pathogen Agrobacterium tumefaciens
Source: Nat Commun. 2022 Dec 24;13:7927. doi: 10.1038/s41467-022-35607-5 (PMC9790009; doi:10.1038/s41467-022-35607-5)
Supplement: Supplementary file 1 — Supplementary Information [file 41467_2022_35607_MOESM1_ESM.pdf]

# Peptidoglycan recycling mediated by an ABC transporter in the plant pathogen *Agrobacterium tumefaciens*

## Supplementary Information

**Michael C. Gilmore, Felipe Cava***

Laboratory for Molecular Infection Medicine Sweden (MIMS), Umeå Center for Microbial Research (UCMR), Department of Molecular Biology, Umeå University, 90187 Umeå, Sweden

*For correspondence: felipe.cava@umu.se

Keywords: bacterial cell wall, peptidoglycan recycling, *Agrobacterium tumefaciens*, Rhizobiales, ABC transporter

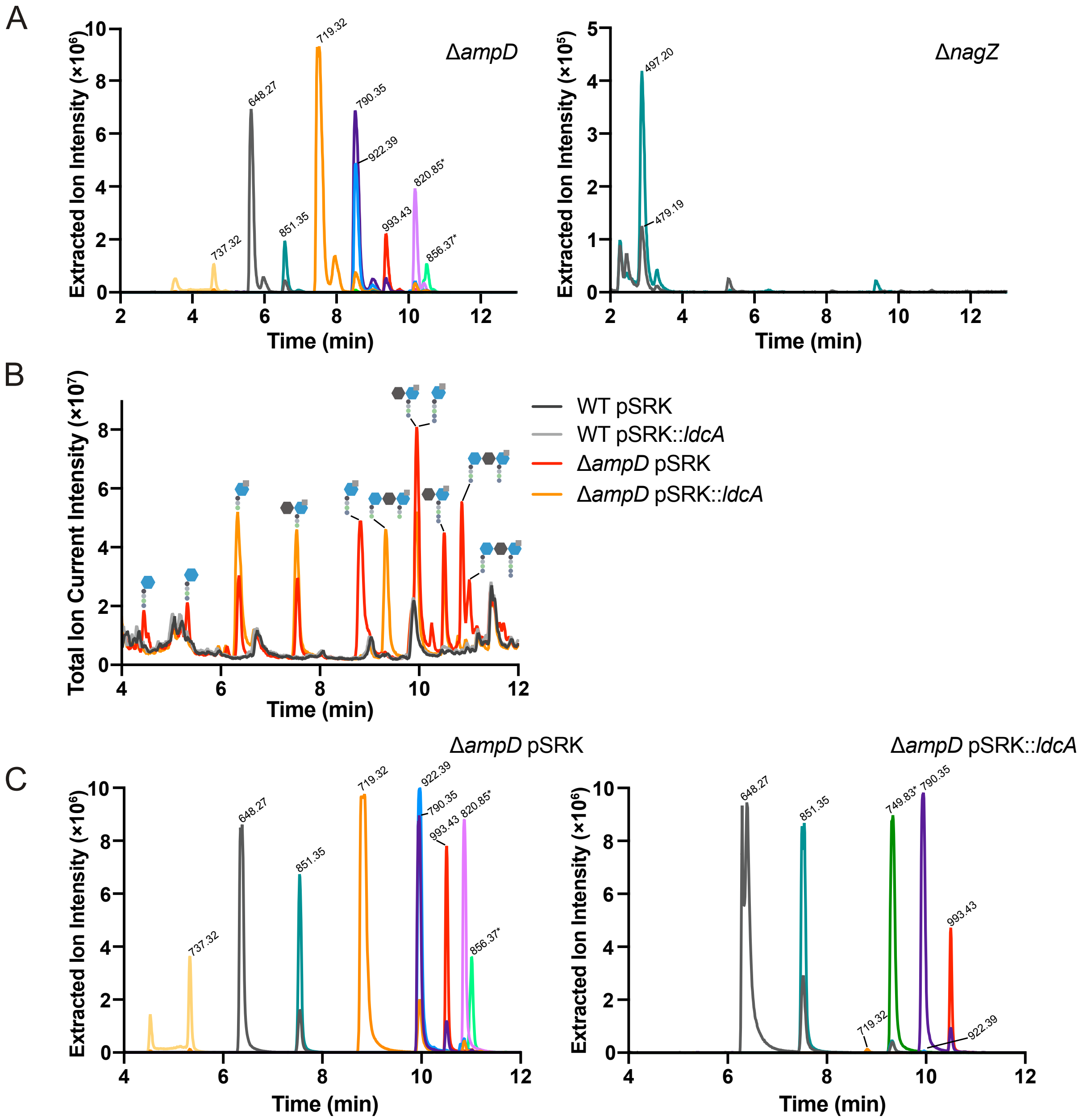


Figure S1. (A). Extracted ion chromatograms showing identified peak *m/z* values of muropeptide species that accumulate in the cytoplasm of *A. tumefaciens* Δ*ampD* and Δ*nagZ* strains. Corresponding muropeptide structures are indicated in Table S3. Single charged $[M+H]^{+}$ ions are shown unless denoted with *, where double charged $[M+2H]^{2+}$ ions are shown as they were more prevalent. (B). Total ion current (TIC) chromatogram showing muropeptide species that accumulate in *A. tumefaciens* WT and Δ*ampD* strains with or without ectopic expression of *E. coli ldcA* detected using UPLC-MS. (C). Extracted ion chromatograms showing identified peak *m/z* values of muropeptide species that accumulate in the cytoplasm of *A. tumefaciens* Δ*ampD* with and without ectopic expression of *E. coli ldcA.*

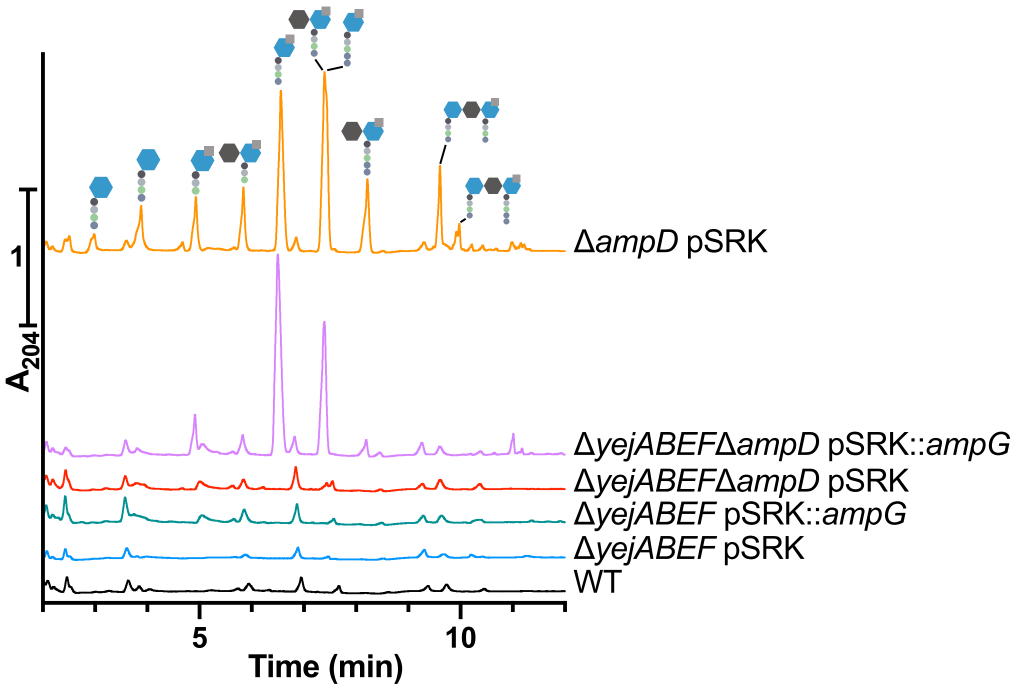


Figure S2. Detection of accumulating muropeptide species in *A. tumefaciens* WT, Δ*ampD*, Δ*yejABEF* and Δ*yejABEF*Δ*ampD* containing expression vector with or without *E. coli ampG* using UPLC. Representative chromatograms from three independent biological replicates are shown.

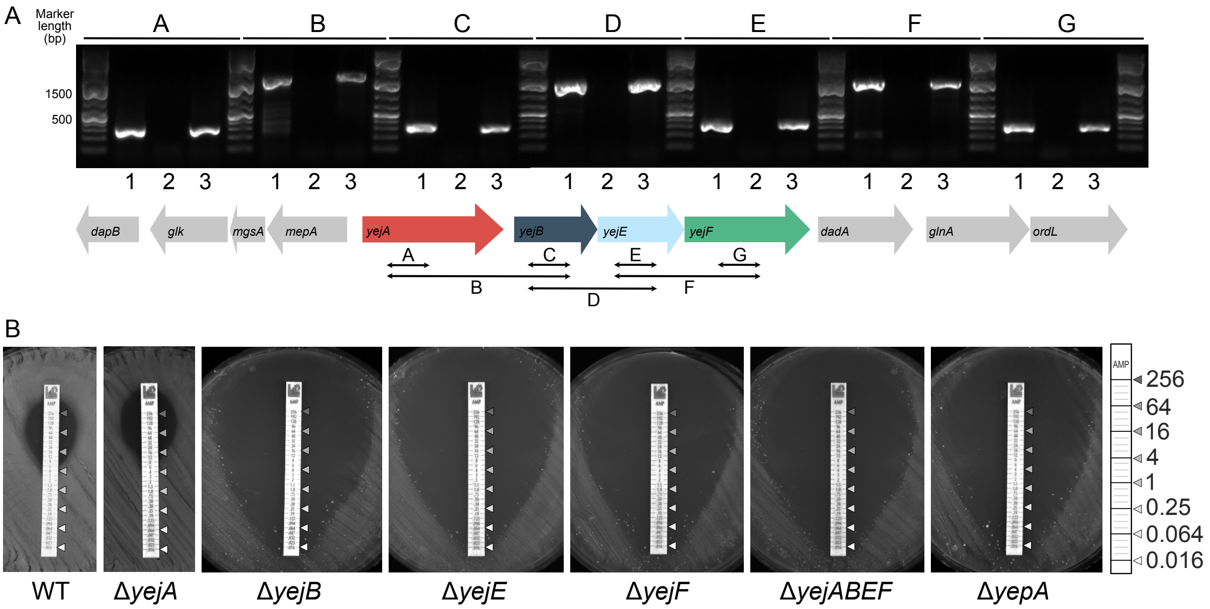


Figure S3. *yejABEF* genes are co-transcribed, but YejA is not involved in PG recycling. (A). PCR amplification using as template: 1. gDNA; 2. RT reaction without added RT; 3. cDNA. Inter- and intragenic regions amplified are indicated below. Results from single RNA extraction are shown. (B). Ampicillin MIC test strips (concentrations in μg/mL) of *A. tumefaciens* WT, Δ*yejA*, Δ*yejB*, Δ*yejE*, Δ*yejF*, Δ*yejABEF* and Δ*yepA* strains.

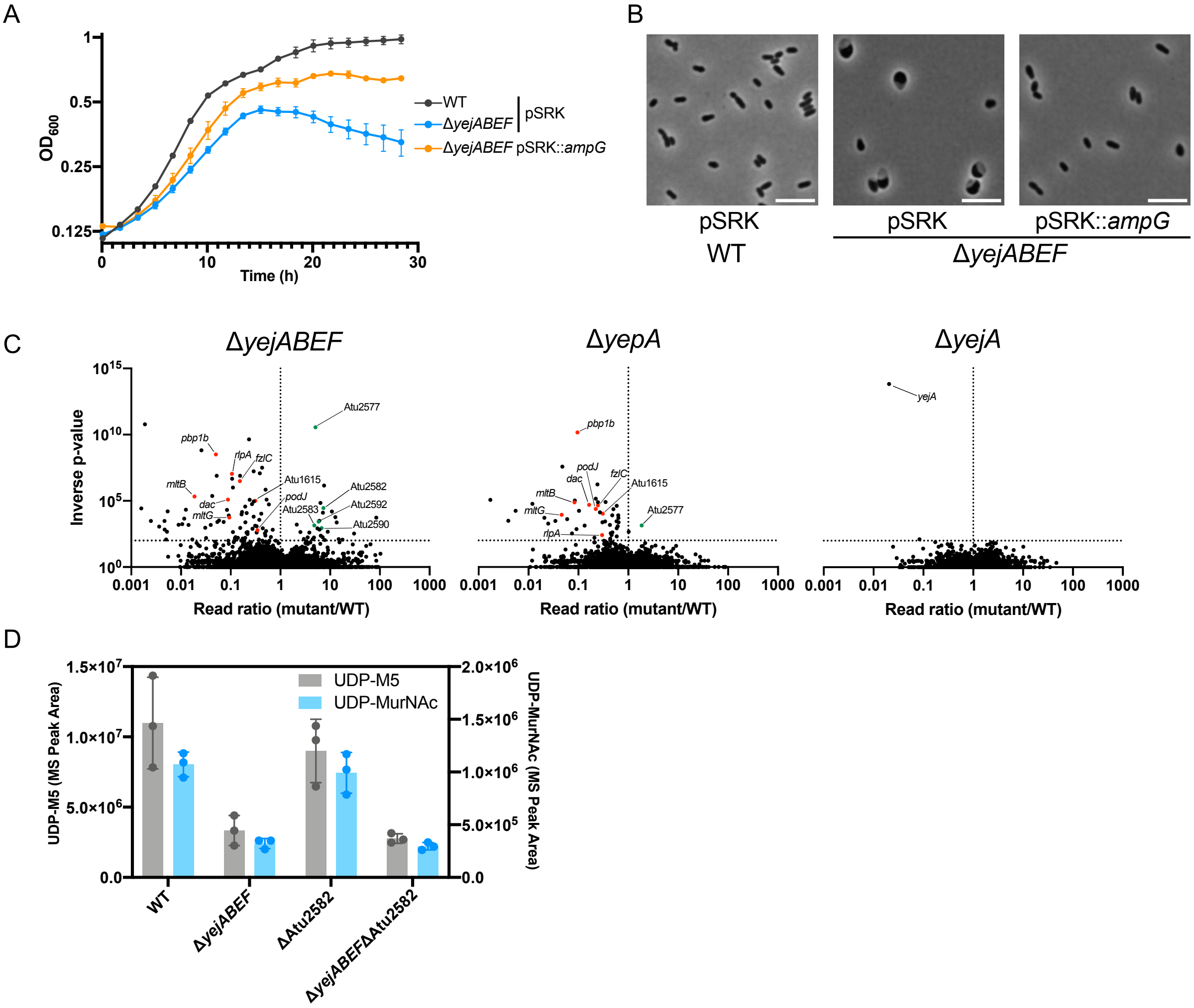


Figure S4. PG recycling is required for cell wall integrity in *A. tumefaciens* (cont.). (A). Growth curves and (B). phase contrast microscopy images of *A. tumefaciens* WT and Δ*yejABEF* with and without ectopic expression of *E. coli ampG* grown in $LB_0$. Scale bar length is 5 μm. Images are representative of three independent biological replicates. (C). Volcano plot showing the ratio of Tn-Seq reads mapped to genes in the *A. tumefaciens* Δ*yejABEF*, Δ*yepA* and Δ*yejA* strains compared to WT as control. $p^{-1}$ value determined from Mann-Whitney U test (threshold $p^{-1} > 100$). Selected synthetically detrimental (red) and synthetically beneficial (green) hits are highlighted. (D). Quantification of PG precursors UDP-M5 and UDP-MurNAc in *A. tumefaciens* WT, Δ*yejABEF*, ΔAtu2582 and Δ*yejABEF*ΔAtu2582 strains using LC-MS. Error bars represent standard deviation from mean.

A

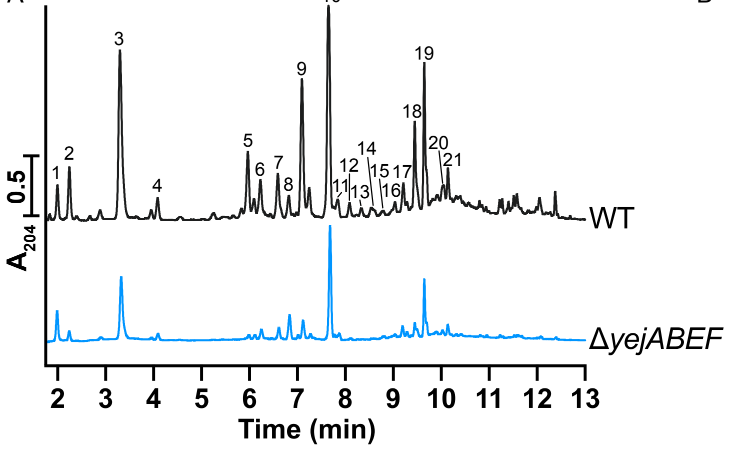


B

| Feature | WT | Δ*yejABEF* |
|---|---|---|
| Tripeptide | 18.43 ± 0.5 | 19.48 ± 0.91 |
| Tetrapeptide | 78.5 ± 0.52 | 77.53 ± 0.49 |
| Pentapeptide | 3.06 ± 0.13 | 2.99 ± 0.47 |
| Monomers | 45.89 ± 2.44 | 50.82 ± 1.06 * |
| Dimers | 38.65 ± 1.01 | 36.69 ± 1.13 |
| Trimers | 13.12 ± 1.53 | 10.92 ± 1.71 |
| Tetramers | 2.34 ± 0.5 | 1.57 ± 0.25 |
| Total crosslinked | 54.11 ± 2.44 | 49.18 ± 1.06 * |
| DD crosslinked | 32.07 ± 1.96 | 40.41 ± 0.72 * |
| LD crosslinked | 22.04 ± 0.49 | 8.78 ± 0.7 * |

C

| | | | MS Analysis | | | Relative Abundance | |
|---|---|---|---|---|---|---|---|
| | Identity | Proposed Structure | Observed *m/z* | Theoretical *m/z* | Difference (mDa) | WT | Δ*yejABEF* |
| 1 | M3 | GlcNAc-MurNAc-L-Ala-D-Glu-m-DAP | 871.3732 | 871.3778 | 4.6 | 3.95 ± 0.27 | 10.71 ± 0.23 * |
| 2 | M4$^{G}$ | GlcNAc-MurNAc-L-Ala-D-Glu-m-DAP-Gly | 928.3992 | 928.3993 | 0.1 | 6.83 ± 0.33 | 3.53 ± 0.2 * |
| 3 | M4 | GlcNAc-MurNAc-L-Ala-D-Glu-m-DAP-D-Ala | 942.4156 | 942.4155 | -0.1 | 32.25 ± 1.86 | 33.67 ± 0.83 |
| 4 | M5 | GlcNAc-MurNAc-L-Ala-D-Glu-m-DAP-D-Ala-D-Ala | 1013.4525 | 1013.4526 | 0.1 | 2.86 ± 0.13 | 2.91 ± 0.56 |
| 5 | D34$^{G}$ | M3-M4$^{G}$ (DAP-DAP crosslink) | 890.8824 | 890.8839 † | 1.5 | 4.70 ± 0.06 | 0.66 ± 0.06 * |
| 6 | D33 | M3-M3 (DAP-DAP crosslink) | 862.3771 | 862.3761† | -1 | 2.71 ± 0.06 | 1.70 ± 0.1 * |
| 7 | D44$^{G}$ | M4-M4$^{G}$ (DAP-D-Ala crosslink) | 926.4043 | 926.4024 † | -1.9 | 3.32 ± 0.11 | 2.12 ± 0.13 * |
| 8 | D43 | M4-M3 (DAP-D-Ala crosslink) | 897.892 | 897.8917 † | -0.3 | 1.08 ± 0.72 | 4.97 ± 0.11 * |
| 9 | D34 | M3-M4 (DAP-DAP crosslink) | 897.8937 | 897.8917 † | -2 | 9.83 ± 0.13 | 3.85 ± 0.18 * |
| 10 | D44 | M4-M4 (DAP-D-Ala crosslink) | 933.4114 | 933.4102 † | -1.2 | 17.01 ± 0.41 | 23.38 ± 1.11 * |
| 11 | T334$^{G}$ | M3-M3-M4$^{G}$ (DAP-DAP crosslinks) | 878.3799 | 878.3785 ‡ | -1.4 | 0.96 ± 0.01 | 0.86 ± 0.29 |
| 12 | T333 | M3-M3-M3 (DAP-DAP crosslinks) | 859.3695 | 859.3714 ‡ | 1.9 | 0.54 ± 0.08 | 0.11 ± 0.1 * |
| 13 | T335$^{G}$ | M3-M3-M5$^{G}$ (DAP-DAP crosslinks) | 902.0539 | 902.0576 ‡ | 3.7 | 0.33 ± 0.02 | 0.00 ± 0 * |
| 14 | T343 | M3-M4-M3 (DAP-DAP, DAP-D-Ala crosslinks) | 883.055 | 883.0504 ‡ | -4.6 | 0.17 ± 0.02 | 0.07 ± 0.12 |
| 15 | T444$^{G}$ | M4-M4-M4 (DAP-D-Ala crosslinks) | 925.737 | 925.7366 ‡ | -0.4 | 0.28 ± 0.03 | 0.25 ± 0.3 |
| 16 | T443 | M4-M4-M3 (DAP-D-Ala crosslinks) | 906.7301 | 906.7295 ‡ | -0.6 | 0.64 ± 0.18 | 0.48 ± 0.41 |
| 17 | T334 | M3-M3-M4 (DAP-DAP, DAP-D-Ala crosslinks) | 883.0495 | 883.0504 ‡ | 0.9 | 1.22 ± 0.22 | 1.39 ± 0.2 |
| 18 | T344 | M3-M4-M4 (DAP-DAP, DAP-D-Ala crosslinks) | 906.7309 | 906.7295 ‡ | -1.4 | 3.77 ± 0.45 | 1.30 ± 0.48 * |
| 19 | T444 | M4-M4-M4 (DAP-D-Ala crosslinks) | 930.4082 | 930.4085 ‡ | 0.3 | 5.21 ± 0.59 | 6.46 ± 0.31 * |
| 20 | Tt4443 | M4-M4-M4-M3 (2×DAP-D-Ala, 1×DAP-DAP crosslinks) | 1214.527 | 1214.5285 ‡ | 1.5 | 1.19 ± 0.23 | 0.60 ± 0.17 * |
| 21 | Tt4444 | M4-M4-M4-M4 (DAP-D-Ala crosslinks) | 1238.2038 | 1238.2075 ‡ | 3.7 | 1.15 ± 0.27 | 0.97 ± 0.09 |

Figure S5. Peptidoglycan composition analysis of *A. tumefaciens* WT and Δ*yejABEF* strains. (A). Representative UPLC chromatograms showing muropeptide profiles obtained from muramidase digestion of purified peptidoglycan (B). Summary of main features of peptidoglycan composition. (C). MS identification and relative quantification of individual muropeptide peaks from UPLC chromatograms. MS *m/z* values represent single charged $[M+H]^+$ ions, except where noted with † ($[M+2H]^{2+}$ ion shown) or ‡ ($[M+3H]^{3+}$ ion shown). * $p < 0.05$ in unpaired two-tailed T-test comparing Δ*yejABEF* results to WT. Quantification values represented as mean of three independent biological replicates ± standard deviation.

# Table S1: Strains

| Bacterial Strain | FC Strain Identifier | Source |
|---|---|---|
| *Agrobacterium tumefaciens* C58 | FC1701 | - |
| *Escherichia coli* DH5α pNPTS139 | FC2289 | Courtesy of P. Brown |
| *Agrobacterium tumefaciens* C58 Δ*ampD* | FC3398 | This study |
| *Agrobacterium tumefaciens* C58 Δ*nagZ* | FC3399 | This study |
| *Escherichia coli* DH5α pSRKpTac | FC2287 | Courtesy of P. Brown |
| *Escherichia coli* DH5α pSRKpTac::*ldcA* | FC3409 | This study |
| *Agrobacterium tumefaciens* C58 pSRKpTac::*ldcA* | FC3411 | This study |
| *Agrobacterium tumefaciens* C58 Δ*ampD* pSRKpTac::*ldcA* | FC3412 | This study |
| *Escherichia coli* SM10 λPIR pSC189 | FC680 | (64) |
| *Agrobacterium tumefaciens* C58 Δ*yejABEF* | FC3400 | This study |
| *Agrobacterium tumefaciens* C58 Δ*yejABEF*Δ*ampD* | FC3401 | This study |
| *Escherichia coli* DH5α pSRKpTac::*ampG* | FC3410 | This study |
| *Agrobacterium tumefaciens* C58 Δ*yejABEF* pSRKpTac::*ampG* | FC3413 | This study |
| *Agrobacterium tumefaciens* C58 Δ*yejABEF*Δ*ampD* pSRKpTac::*ampG* | FC3414 | This study |
| *Agrobacterium tumefaciens* C58 Δ*ampC* | FC3402 | This study |
| *Agrobacterium tumefaciens* C58 Δ*yejABEF*Δ*ampC* | FC3403 | This study |
| *Agrobacterium tumefaciens* C58 Δ*yejA* | FC3404 | This study |
| *Agrobacterium tumefaciens* C58 Δ*yejB* | FC3405 | This study |
| *Agrobacterium tumefaciens* C58 Δ*yejE* | FC3406 | This study |
| *Agrobacterium tumefaciens* C58 Δ*yejF* | FC3407 | This study |
| *Agrobacterium tumefaciens* C58 Δ*yepA* | FC3408 | This study |

# Table S2: Primers

| Primer | FCP Identifier | Sequence (5’ -> 3’) |
|---|---|---|
| ampD_UF | FCP3880 | GGCCAAGCTTCGCCCGTCTTTATAACCTCG |
| ampD_UR | FCP3881 | GAAATGTCGCTGGAAGGCAGGGGCAAAATCCGGCAGACAT |
| ampD_DF | FCP3882 | ATGTCTGCCGGATTTTGCCCCTGCCTTCCAGCGACATTT |
| ampD_DR | FCP3883 | GGCCGAATTCCACGAAGGTCTCCAGCGTCA |
| nagZ_UF | FCP3907 | GGCCAAGCTTGACATCAAGGCCGCCGAAAT |
| nagZ_UR | FCP3908 | CTTCAGCGCATTCATGGAGCATGAAACCCCAGGGCTGCTC |
| nagZ_DF | FCP3909 | GAGCAGCCCTGGGGTTTCATGCTCCATGAATGCGCTGAAG |
| nagZ_DR | FCP3910 | GGCCGAATTCAATACGCGACAGATCGACCT |
| yejABEF_UF | FCP4525 | AACCGGATCCCCAGCCGATCTTCTGCGCAT |
| yejABEF_UR | FCP4526 | ATTGAAGGCCGCTGCCAGCAGTTGGTGCCAATATCATCGGGG |
| yejABEF_DF | FCP4527 | CCCCGATGATATTGGCACCAACTGCTGGCAGCGGCCTTCAAT |
| yejABEF_DF | FCP4528 | AACCGTCGACTGCCTGATGGCTGCGGAATT |
| yejA_UR | FCP4529 | GATGAACATCTCGCCACGGTGGTGCCAATATCATCGGGG |
| yejA_DF | FCP4530 | CCCCGATGATATTGGCACCAACCGTGGCGAGATGTTCATC |
| yejA_DR | FCP4531 | AACCGTCGACATCGAAACCACCGGACTGGC |
| pSRK_ampG_F_NdeI | FCP4634 | CTTCATATGTCCAGTCAATATTTACGTATT |
| pSRK_ampG_R_HindIII_stop | FCP4783 | CTTAAGCTTTCACGTCAGATGCGTTTTTCGTA |
| ampC_U | FCP4630 | AACCGGATCCCCACGACCCCGAGAAACAGCAA |
| ampC_UR | FCP4631 | AGAGACGCGTTCCTCATTGGCCAAAGCGGCCAGTGCGATA |
| ampC_DF | FCP4632 | TATCGCACTGGCCGCTTTGGCCAATGAGGAACGCGTCTCT |
| ampC_DR | FCP4633 | AACCGTCGACGTAGAACCGAACATGGCCAC |
| pSRK_ldcA_F_NdeI | FCP4745 | GAACATATGTCTCTGTTTCACTTAATTGC |
| pSRK_ldcA_R_HindIII | FCP4746 | GAAAAGCTTTTACATTTTAAGAACAGGATG |
| yejB_UF | FCP4892 | GCGCCGGCCAGGCGCCAGAAGGCAACGAACAGGCGGATTT |
| yejB_UR | FCP4893 | AATCGATGCGCGGATCGATCCCGATCGTCGGGATCATCAGT |
| yejB_DF | FCP4894 | GGATCGATCCGCGCATCGAT |
| yejB_DR | FCP4895 | CGCGTTCGGCCGTGCTAGCGTTGCCAAGCGTACAGCCGGG |
| yejE_UF | FCP4273 | AACCGGATCCACCAGCTGAACTGGTGGCAGAAGAT |
| yejE_UR | FCP4274 | ATCTCACCCAGAGACGGCGATCTTACCAGCGTTTCGGTTCCGGC |
| yejE_DF | FCP4275 | GCCGGAACCGAAACGCTGGTAAGATCGCCGTCTCTGGGTGAGAT |
| yejE_DR | FCP4276 | AACCGTCGACATGGTGATGTCGTTGCCGCG |
| yejF_UF | FCP4888 | GCGCCGGCCAGGCGCCAGAAACCTGCTTTTGCAGCGTTTC |
| yejF_UR | FCP4889 | GTGTAATCCTGCTGCGGATTATCGACAGCGACGCTGGTTG |
| yejF_DF | FCP4890 | AATCCGCAGCAGGATTACAC |
| yejF_DR | FCP4891 | CGCGTTCGGCCGTGCTAGCGACACGGAGTGCTCGATCAGC |
| yepA_UF | FCP5281 | TCCTGCAGGATATCGTGGATCCAGGTCTTGAACCAGCCGAGC |
| yepA_UR | FCP5282 | AATGTAGTGGCCGGCCACGAGCATGGATATGCCGTGCAGCG |
| yepA_DF | FCP5283 | CTCGTGGCCGGCCACTACAT |
| yepA_DR | FCP5284 | AATACGACTCACTAGTGGGTCGACTTTCCTGAACGTCGCGGCCT |
| yejA_coexp_F | FCP6349 | GACGGCAAGCCGCGTGACATAT |
| yejA_coexp_R | FCP6350 | TTAACGCGGCCTTCCTTCACGG |
| yejB_coexp_F | FCP6351 | CGCTCACCCGTTTTCTTGAA |
| yejB_coexp_R | FCP6352 | GTCGAAGAATGATCCGCCGG |
| yejE_coexp_F | FCP6353 | GGATGAGATCAACGCCAATG |
| yejE_coexp_R | FCP6354 | AAATACCCCTGAATGGCGCC |
| yejF_coexp_F | FCP6355 | CTGCTGTTGCAGGTCGGCAT |
| yejF_coexp_R | FCP6356 | AACGATCTTGCCCTTGGTCA |

# Table S3: Identified Muropeptides

| Schematic | Name | Composition | Ion $[M+H]^+$ | | Difference |
|---|---|---|---|---|---|
| | | | Observed | Expected | (mDa) |
| 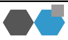 | DSN | GlcNAc-anhydroMurNAc | 479.1876 | 479.1872 | -0.4 |
| 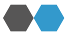 | DS | GlcNAc-MurNAc | 497.1990 | 497.1977 | -1.3 |
| 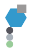 | anhMurNAc-P3 | anhydroMurNAc-L-Ala-D-Glu-m-DAP | 648.2722 | 648.2723 | 0.1 |
| 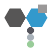 | M3N | GlcNAc-anhydroMurNAc-L-Ala-D-Glu-m-DAP | 851.3515 | 851.3517 | 0.2 |
| 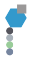 | anhMurNAc-P4 | anhydroMurNAc-L-Ala-D-Glu-m-DAP-D-Ala | 719.3102 | 719.3094 | -0.8 |
| 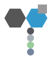 | M4N | GlcNAc-anhydroMurNAc-L-Ala-D-Glu-m-DAP-D-Ala | 922.3877 | 922.3888 | 1.1 |
| 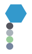 | MurNAc-P5 | MurNAc-L-Ala-D-Glu-m-DAP-D-Ala | 737.3199 | 737.3200 | 0.1 |
| 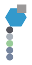 | anhMurNAc-P5 | anhydroMurNAc-L-Ala-D-Glu-m-DAP-D-Ala-D-Ala | 790.3455 | 790.3465 | 1 |
| 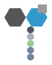 | M5N | GlcNAc-anhydroMurNAc-L-Ala-D-Glu-m-DAP-D-Ala-D-Ala | 993.4251 | 993.4259 | 0.8 |
| 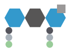 | M3-M3N | MurNAc-(L-Ala-D-Glu-m-DAP)-GlcNAc-anhydroMurNAc-L-Ala-D-Glu-m-DAP | 1498.6155 | 1498.6166 | 1.1 |
| 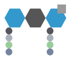 | M4-M4N | MurNAc-(L-Ala-D-Glu-m-DAP-D-Ala)-GlcNAc-anhydroMurNAc-L-Ala-D-Glu-m-DAP-D-Ala | 1640.6883 | 1640.6909 | 2.6 |
| 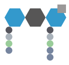 | M4-M5N | MurNAc-(L-Ala-D-Glu-m-DAP-D-Ala)-GlcNAc-anhydroMurNAc-L-Ala-D-Glu-m-DAP-D-Ala-D-Ala | 1711.7255 | 1711.7280 | 2.5 |
| 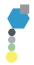 | anhMurNAc-$P4^{Met}$ | anhydroMurNAc-L-Ala-D-Glu-m-DAP-D-Met | 779.3111 | 779.3128 | 1.7 |
| 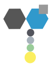 | $M4N^{Met}$ | GlcNAc-anhydroMurNAc-L-Ala-D-Glu-m-DAP-D-Met | 982.3940 | 982.3921 | -1.9 |
| 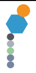 | UDP-M5 | Uridine-diphosphate-MurNAc-L-Ala-D-Glu-m-DAP-D-Ala-D-Ala | 1194.3491 | 1194.3487 | -0.4 |
