# Supplementary material for: Peptidoglycan recycling mediated by an ABC transporter in the plant pathogen Agrobacterium tumefaciens
Source: Nat Commun. 2022 Dec 24;13:7927. doi: 10.1038/s41467-022-35607-5 (PMC9790009; doi:10.1038/s41467-022-35607-5)
Supplement: Supplementary file 3 — Description of Additional Supplementary Files [file 41467_2022_35607_MOESM3_ESM.pdf]

Description of Additional Supplementary Files:

Supplementary Data 1. Tn-Seq screen in A. tumefaciens WT with 2 mg/mL Fosfomycin

Supplementary Data 2. Tn-Seq screen in A. tumefaciens ΔyejABEF, ΔyepA and ΔyejA strains compared to WT
