# Supplementary material for: Peptidoglycan recycling mediated by an ABC transporter in the plant pathogen Agrobacterium tumefaciens
Source: Nat Commun. 2022 Dec 24;13:7927. doi: 10.1038/s41467-022-35607-5 (PMC9790009; doi:10.1038/s41467-022-35607-5)
Supplement: Supplementary file 6 — Reporting Summary [file 41467_2022_35607_MOESM6_ESM.pdf]

nature portfolio

Corresponding author(s): Felipe Cava

Last updated by author(s): Dec 6, 2022

Reporting Summary

Nature Portfolio wishes to improve the reproducibility of the work that we publish. This form provides structure for consistency and transparency in reporting. For further information on Nature Portfolio policies, see our Editorial Policies and the Editorial Policy Checklist.

Statistics

For all statistical analyses, confirm that the following items are present in the figure legend, table legend, main text, or Methods section.

n/a	Confirmed	
☐	☒	The exact sample size (*n*) for each experimental group/condition, given as a discrete number and unit of measurement
☒	☐	A statement on whether measurements were taken from distinct samples or whether the same sample was measured repeatedly
☐	☒	The statistical test(s) used AND whether they are one- or two-sided<br>*Only common tests should be described solely by name; describe more complex techniques in the Methods section.*
☒	☐	A description of all covariates tested
☒	☐	A description of any assumptions or corrections, such as tests of normality and adjustment for multiple comparisons
☐	☒	A full description of the statistical parameters including central tendency (e.g. means) or other basic estimates (e.g. regression coefficient) AND variation (e.g. standard deviation) or associated estimates of uncertainty (e.g. confidence intervals)
☐	☒	For null hypothesis testing, the test statistic (e.g. *F*, *t*, *r*) with confidence intervals, effect sizes, degrees of freedom and *P* value noted<br>*Give P values as exact values whenever suitable.*
☒	☐	For Bayesian analysis, information on the choice of priors and Markov chain Monte Carlo settings
☒	☐	For hierarchical and complex designs, identification of the appropriate level for tests and full reporting of outcomes
☒	☐	Estimates of effect sizes (e.g. Cohen's *d*, Pearson's *r*), indicating how they were calculated

Our web collection on statistics for biologists contains articles on many of the points above.

Software and code

Policy information about availability of computer code

Data collection	No custom code was generated for this study. For data collection, the following software was used: BioTek Gen5 3.09, Waters UNIFI 1.8.1, Waters Empower 3.6
Data analysis	No custom code was generated for this study. For data analysis, the following software was used: ACD/Labs ChemSketch 2012, Waters UNIFI 1.8.1, Waters Empower 3.6, eggNOG 5, MUSCLE 5, JDet 1.4.9, WebLogo 3, iTOL 6

For manuscripts utilizing custom algorithms or software that are central to the research but not yet described in published literature, software must be made available to editors and reviewers. We strongly encourage code deposition in a community repository (e.g. GitHub). See the Nature Portfolio guidelines for submitting code & software for further information.

Data

Policy information about availability of data

All manuscripts must include a data availability statement. This statement should provide the following information, where applicable:

- Accession codes, unique identifiers, or web links for publicly available datasets
- A description of any restrictions on data availability
- For clinical datasets or third party data, please ensure that the statement adheres to our policy

Raw sequencing reads for Tn-Seq experiments are available at NCBI Sequence Read Archive (BioProjects PRJNA869898 and PRJNA869899). Sequences for YejA and AmpG orthologs were obtained from OrthoDB v10, groups 231800at1224 for YejA and 542870at1224 for AmpG. Reference AmpG and SBP sequences were obtained from UniProt under accession codes P0AE16 for AmpG and as follows for SBPs: A. tumefaciens (YejA: A9CKL4, YepA: A9CIN5), Sinorhizobium meliloti (YejA:

Q92T30, YepA: Q92PT7), Bartonella henselae (YepA only: A0A0H3LXD4), Brucella melitensis (YejA: Q8YEE4, YepA: Q8YC41), Ochrobactrum anthropi (YejA: A6WUU2, YepA: A6X540), Dinoroseobacter shibae (YejA: A8LQB5, YepA: A8LK88) and Phaeobacter inhibens (YejA: I7EQS1, YepA: I7EWM7). All other data generated or analysed during this study are included in this published article, its supplementary information and Source Data files.

Human research participants

Policy information about studies involving human research participants and Sex and Gender in Research.

Reporting on sex and gender	N/A
Population characteristics	N/A
Recruitment	N/A
Ethics oversight	N/A

Note that full information on the approval of the study protocol must also be provided in the manuscript.

Field-specific reporting

Please select the one below that is the best fit for your research. If you are not sure, read the appropriate sections before making your selection.

[x] Life sciences [ ] Behavioural & social sciences [ ] Ecological, evolutionary & environmental sciences

For a reference copy of the document with all sections, see nature.com/documents/nr-reporting-summary-flat.pdf

Life sciences study design

All studies must disclose on these points even when the disclosure is negative.

Sample size	Three biological replicates were used to account for random variation. A sample size of three was chosen because it provided sufficient statistical power to rule out differences due to inherent biological variation.
Data exclusions	No data were excluded
Replication	Three biological replicates were used for each experiment, with the results shown as separate points on bar charts. The only exception is the M4NMet incorporation experiment (Fig. 1E), where only one biological replicate was performed due to low amounts of available M4NMet. Where chromatograms or microscopy images are shown, these are representative of three biological replicates
Randomization	Experimental group allocation is not relevant due to the molecular biology nature of our study
Blinding	We deemed blinding of experiments unnecessary due to the low chance for bias to influence results due to the molecular biology nature of our study.

Reporting for specific materials, systems and methods

We require information from authors about some types of materials, experimental systems and methods used in many studies. Here, indicate whether each material, system or method listed is relevant to your study. If you are not sure if a list item applies to your research, read the appropriate section before selecting a response.

Materials & experimental systems

n/a	Involved in the study	
[x]	[ ]	Antibodies
[x]	[ ]	Eukaryotic cell lines
[x]	[ ]	Palaeontology and archaeology
[x]	[ ]	Animals and other organisms
[x]	[ ]	Clinical data
[x]	[ ]	Dual use research of concern

Methods

n/a	Involved in the study	
[x]	[ ]	ChIP-seq
[x]	[ ]	Flow cytometry
[x]	[ ]	MRI-based neuroimaging
